# MiR171h restricts root symbioses and shows like its target *NSP2* a complex transcriptional regulation in *Medicago truncatula*

**DOI:** 10.1186/s12870-014-0199-1

**Published:** 2014-07-23

**Authors:** Vinzenz Hofferek, Amelie Mendrinna, Nicole Gaude, Franziska Krajinski, Emanuel A Devers

**Affiliations:** 1 Max-Planck-Institute of Molecular Plant Physiology, Am Mühlenberg 1, Potsdam, 14476, (OT) Golm, Germany; 2 Present address: Department of Biology, Swiss Federal Institute of Technology Zurich, Zürich, Switzerland

**Keywords:** Symbiosis, Plant miRNA, miR171h, NSP2, Plant nutrition

## Abstract

**Background:**

Legumes have the unique capability to undergo root nodule and arbuscular mycorrhizal symbiosis. Both types of root endosymbiosis are regulated by *NSP2*, which is a target of microRNA171h (miR171h). Although, recent data implies that miR171h specifically restricts arbuscular mycorrhizal symbiosis in the root elongation zone of *Medicago truncatula* roots, there is limited knowledge available about the spatio-temporal regulation of miR171h expression at different physiological and symbiotic conditions.

**Results:**

We show that miR171h is functionally expressed from an unusual long primary transcript, previously predicted to encode two identical miR171h strands. Both miR171h and *NSP2* transcripts display a complex regulation pattern, which involves the symbiotic status and the fertilization regime of the plant. Quantitative Real-time PCR revealed that miR171h and *NSP2* transcript levels show a clear anti-correlation in all tested conditions except in mycorrhizal roots, where *NSP2* transcript levels were induced despite of an increased miR171h expression. This was also supported by a clear correlation of transcript levels of *NSP2* and *MtPt4*, a phosphate transporter specifically expressed in a functional AM symbiosis. MiR171h is strongly induced in plants growing in sufficient phosphate conditions, which we demonstrate to be independent of the *CRE1* signaling pathway and which is also not required for transcriptional induction of *NSP2* in mycorrhizal roots. *In situ* hybridization and promoter activity analysis of both genes confirmed the complex regulation involving the symbiotic status, P and N nutrition, where both genes show a mainly mutual exclusive expression pattern. Overexpression of miR171h in *M. truncatula* roots led to a reduction in mycorrhizal colonization and to a reduced nodulation by *Sinorhizobium meliloti*.

**Conclusion:**

The spatio-temporal expression of miR171h and *NSP2* is tightly linked to the nutritional status of the plant and, together with the results from the overexpression analysis, points to an important function of miR171h to integrate the nutrient homeostasis in order to safeguard the expression domain of *NSP2* during both, arbuscular mycorrhizal and root nodule symbiosis.

## Background

Plants constantly have to cope with phosphate (P_i_) limiting conditions and one strategy to overcome P_i_ limitation is the development of a mutualistic association called arbuscular mycorrhizal symbiosis (AMS), which is formed by most land plants and fungi of the phylum *Glomeromycota*. AMS can enhance phosphate uptake and growth of the plant [[1],[2]] and is named for the formation of intracellular tree like structures called arbuscules. Arbuscules are mostly formed in the inner cortical cell layer of mycorrhizal roots and are always surrounded by the plant-derived periarbuscular membrane (PAM), the site of nutrient exchange [[3],[4]]. The formation of AMS is initiated after a chemical dialogue between the host plant and symbiont [[5]]. The plant secretes strigolactones, a group of plant hormones known to stimulate of fungal spore germination and hyphal branching [[6]]. In return, the fungus releases a complex mixture of lipochito-oligosaccharides, called Myc-LCOs or Myc-factors, which stimulate the formation of AMS and induces lateral root formation in the legume plant *Medicago truncatula* [[7]].

Strigolactone synthesis and secretion is induced by P_i_ limitation [[8]–[11]]. Biosynthesis of strigolactones requires the GRAS type transcription factors N*odulation* S*ignaling* P*athway* (NSP) *1* and *NSP2*, and *nsp1nsp2* double mutants show a reduced colonization by mycorrhizal fungi [[12]]. Interestingly, *NSP1* and *NSP2* are key components of the Nod-factor signaling pathway leading to root nodule formation [[13],[14]]. Recent results [[7]] suggest that *NSP2* is also involved in AMS. The authors showed that *nsp2* mutant plants do not respond to Myc-LCOs and are less colonized. Furthermore, NSP2 interacts with another GRAS transcription factor *R*equired for *A*rbuscular *M*ycorrhization (RAM) 1, and controls the expression of the glycerol-3-phosphate acyltransferase *RAM2*. Mutations in both genes lead to strongly reduced colonization by *Rhizophagus irregularis.* Interestingly, colonization with the pathogenic oomycete *Phytophthora palmivora* is also impaired in ram1 and ram2 mutants (if otherwise, need to specify) [[15],[16]]. The important role of *NSP2* in strigolactone biosynthesis, RNS and AMS implies that *NSP2* is an integral component of the common signaling pathway [[17]]. Therefore, it can be expected that the spatial and temporal expression of *NSP2* is tightly controlled.

Micro RNAs (miRNAs) are key regulators of gene expression and act by target transcript cleavage and/or translational repression [[18],[19]]. These small non-coding RNA molecules are predominantly 21 nt in size and have an important role in regulating developmental processes, hormonal signaling, organ polarity, RNA metabolism, and abiotic and biotic stresses of the plant [[20]–[24]]. Some miRNAs, e.g. miR166 and miR169, have also been found to be involved in root nodule symbiosis [[25]–[27]]. First evidence that miRNAs are also involved in AMS came from the observation that multiple miRNAs, e.g. miR399, are differentially regulated in the shoots and roots of mycorrhizal *M. truncatula,* tobacco and tomato plants [[28],[29]]. Cloning and deep sequencing of the small RNAs and the degradome of mycorrhizal *M. truncatula* roots identified many miRNAs and their target mRNAs, of which several were differentially expressed in mycorrhizal roots [[30]]. Interestingly, a novel member of the miRNA171 family, miR171h, was shown to target *NSP2* [[30],[31]]. Given the above-mentioned role of *NSP2*, miR171h has been implicated in regulating root endosymbiosis by controlling a key component of the Sym-pathway [[27]]. This assumption has been strengthened by showing miRNA171h expression affects the mycorrhizal colonization, is induced by Myc-LCOs, and is conserved among mycotrophic plants [[32]]. Also, it was shown that the expression of both, miR171h and *NSP2*, is induced upon cytokinin treatment and that this regulation is dependent on Cytokinin Response1 (CRE1) [[33]]. Cytokinins and *CRE1* are involved in nodule organogenesis [[34]], but cytokinins have also been implicated to be involved in arbuscular mycorrhizal symbiosis [[35]]. Additionally, a recent study employing deep sequencing of *Lotus japonicus* nodules revealed a non-canonical miR171 isoform, related to *Medicago* miR171h, which targets *LjNSP2* [[36]]. These results indicate an additional regulating role of miR171h in nodule symbiosis, however a direct involvement could not be demonstrated so far [[32],[36]].

In this study we show that miR171h negatively regulates both types of root endo-symbioses through perception of the nutritional status of the host plant and shows a mutually exclusive expression pattern with its target *NSP2* in the root cortex of *M. truncatula* plants.

## Results

### Expression of an 811 bp miR171h primary transcript mediates NSP2 transcript cleavage in vivo

**Figure 1:**
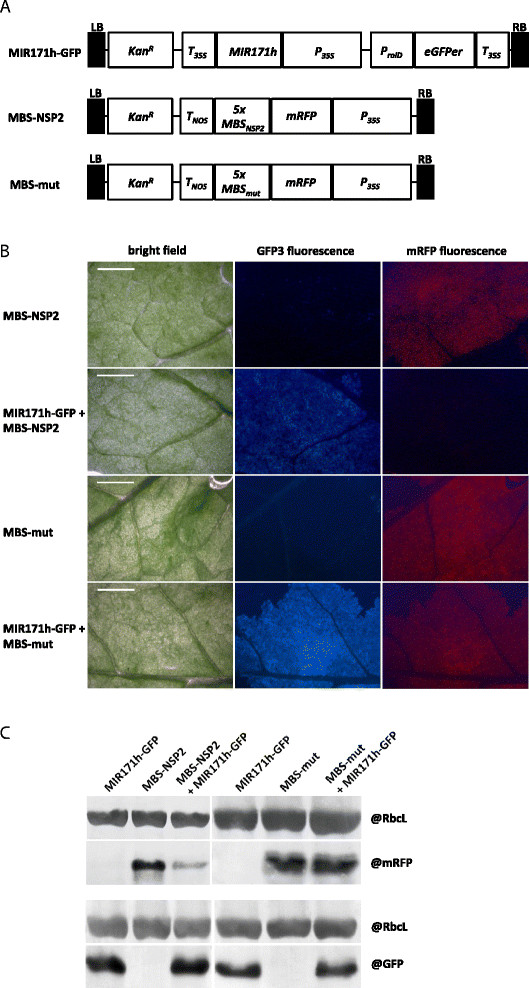
***In vivo*** **confirmation of** ***NSP2*** **gene silencing by miR171h using MIR171h overexpression and mRFP sensor constructs. (A)** T-DNA structure of vectors used for leave infiltration experiments. MiR171h overexpression construct (MIR171h-GFP) in pK7WG2D [[37]] and sensor constructs with either wild-type (MBS-NSP2) or mutated miR171h (MBS-mut) binding site of NSP2 cloned in pGWB455 [[38]]. LB: left boarder, Kan^R^: kanamycin resistance gene (nptII), Tnos: nopaline synthase terminator, MIR171h: miR171h primary transcript, P_35S_: 35S promoter, green-fluorescent protein (GFP) cassette (pRolD–EgfpER–t35S), 5xMBS_NSP2_: 5 repeats of miR171h binding site sequence of NSP2, 5xMBS_mut_: 5 repeats of a mutated version of the miR171h binding site sequence of NSP2. **(B)** Co-infiltration of miR171h overexpression and mRFP sensor constructs. *Nicotiana benthamiana* leaves were infiltrated with the two sensor constructs MBS-NSP2 or MBS-mut. For each sensor construct, co-infiltration experiments with the MIR171h-GFP construct were carried out. Note the decreased mRFP fluorescence due to miR171h-mediated cleavage of mRFP sensor. Bright field images, GFP3 fluorescence and mRFP fluorescence are shown. Scale bar: 5 mm. **(C)** Western blot to prove miR171h cleavage of the miR171h binding site within the NSP2 sequence. Proteins were extracted from leaves infiltrated with MIR171h-GFP, MBS-NSP2 or MBS-mut and co-infiltration of both constructs. The upper part of the picture shows a western blot where mRFP was detected, indicating the presence of the sensor; the lower part shows a western blot with detection of GFP, indirectly indicating the presence of miR171h. On both blots, RuBisCO proteins were detected to demonstrate equal loading of the protein samples.

To confirm the functionality of the predicted 811 bp primary transcript of miR171h [[31]] and the potential to silence *NSP2 in vivo*, we applied miRNA sensor constructs. Three different constructs were applied (Figure 1A). The first construct, MIR171h-GFP, contained an 811 bp fragment of the MIR171h primary transcript [[31]], which was constitutively expressed by a 35S-promoter. The construct includes an independent and constitutively expressed eGFPer as a visual transformation control. The second construct, *m*iR171h *b*inding *s*ite (MBS)-NSP2, represented the actual miRNA sensor. It was composed of a 35S-promoter driven mRFP fused to five repeats of the miR171h binding site of *NSP2*. As a control, the MBS of *NSP2* was mutated to a scrambled sequence (MBS-mut), which was unable to bind miR171h. The constructs were used in *Agrobacterium tumefaciens*-mediated tobacco leaf infiltration assays and the mRFP fluorescence around the infiltration site was monitored (Figure 1B and Additional file 1: Figure S1). When MBS-NSP2 was co-infiltrated with MIR171H-GFP, the mRFP fluorescence was abolished. The fluorescence was restored when MBS-mut and MIR171H-GFP were co-infiltrated, indicating that the loss of mRFP fluorescence was specifically due to miR171h-mediated sensor cleavage. The loss of fluorescence was due to drastically reduced mRFP protein levels in MIR171H-GFP co-infiltrated tobacco leaves of MBS-NSP2 compared to MBS-mut and single infiltration (Figure 1C and Additional file 1: Figure S1). These results confirmed that *NSP2* is regulated by miR171h through specific binding of this miRNA to its previously identified binding site and is consistent with previous degradome results [[30]] and RACE experiments [[32]].

### MiR171h and NSP2 transcript levels are affected by the symbiotic status of the root and by P and N levels

**Figure 2:**
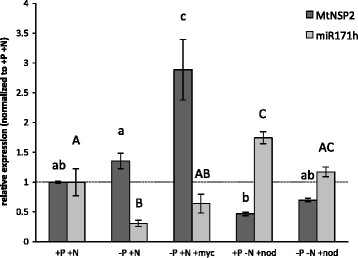
**Relative expression levels of mature miR171h and** ***NSP2*** **transcripts in** ***M. truncatula*** **roots.** -P: 20 μM phosphate, +P: 1 mM phosphate, −N: 0 mM; +N 5 mM nitrate fertilization, myc: mycorrhizal roots, nod: nodulated by *Sinorhizobium meliloti*. Plants were harvested 3 wpi. Normalization of the expression data was carried out against a reference gene index (MtPdf2 and MtEf1) and the resulting relative expression was normalized to full nutrition condition (+P + N). Data shown are average values of 3–4 biological with two technical replicates each. Error bars indicate the standard errors. Different letters indicate statistical different values (P < 0.05, two-way ANOVA with Holm-Sidak multiple comparison).

Previous studies suggested that miR171h is induced in the root elongation zone of mycorrhizal roots and that *NSP2* transcript levels are slightly repressed in mycorrhizal roots [[32]]. Also, miR171h transcription is directly induced by high phosphate nutrition [[30]]. To investigate the transcriptional regulation of *NSP2* and miR171h in more detail, we analyzed both transcript levels in response to mycorrhizal symbiosis and root nodulation, and under different phosphate and nitrogen fertilization treatments. For this purpose we compared the abundance of mature miR171h to the relative transcript abundance of *NSP2* after normalization to full nutrition (+P + N) conditions (Figure 2). As expected, miR171h accumulation was repressed by phosphate starvation (P ≤ 0.05), i.e. positively influenced by high phosphate conditions. It also increases in nodulated roots, in which case it is further enhanced by nitrogen starvation. These results indicate that both *NSP2* and MIR171h show a complex regulation of their transcription, which depends on the symbiotic status of the roots and the nutrient fertilization regime.

### NSP2 transcript levels in mycorrhizal roots are elevated despite of increased miR171h expression

A clear anti-correlation (r = −0.98; p < 0.05) of miR171h and *NSP2* accumulation was present in all but the mycorrhizal condition (Figure 2). In the conditions used, *NSP2* shows the highest relative transcript abundance in mycorrhizal roots as compared to all other conditions tested.

To investigate the miR171h and *NSP2* expression in mycorrhizal roots over a time-course of AM symbiosis development, an experiment of 6 weeks was carried out and RNA accumulation of marker genes for AM symbiosis development and function were analyzed (Additional file 1: Figure S2). This clearly showed that the expression of pri-miR171h increases in mycorrhizal roots from 2 weeks post inoculation on, as compared to non-mycorrhizal roots under the applied conditions (−P, +N). Additionally, the time-course confirms elevated *NSP2* transcript levels in mycorrhizal roots, despite of enhanced miR171h accumulation. Therefore we assume that the abundance of *NSP2* transcript levels in mycorrhizal roots is maintained by a miR171h-independent factor.

**Figure 3:**
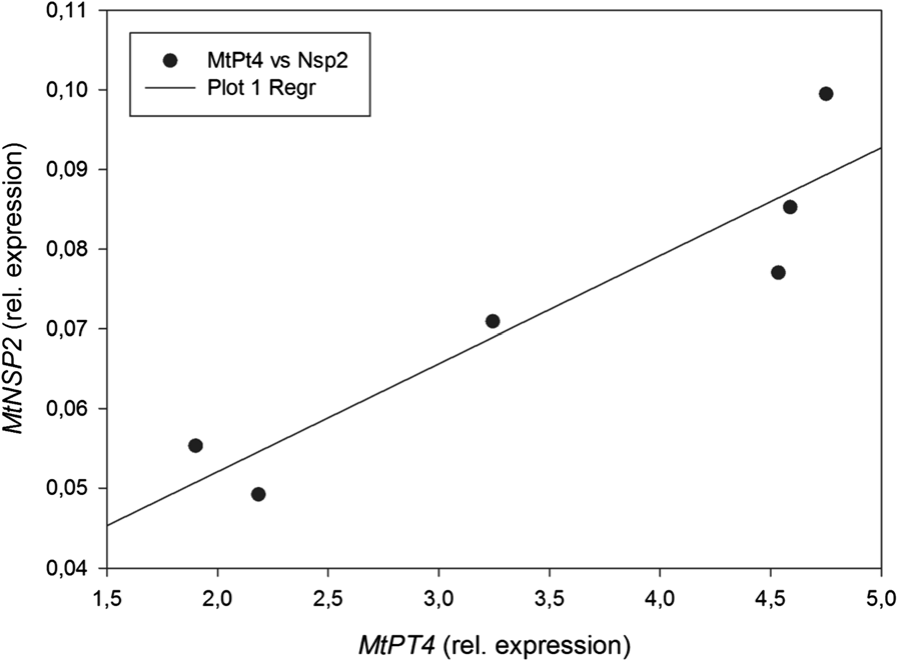
**The relative transcript abundance of** ***NSP2*** **positively correlates with MtPt4.** Scatter plot of the relative expression of *NSP2* against the relative expression of MtPt4 of individual WT plants including a linear regression (black line). A statistically significant correlation was calculated (r = 0.927, P <0.01, Pearson product moment correlation). All plants were harvested 4 wpi. Normalization of the expression data was carried out against a reference gene index (*MtPdf2* and *MtEf1*).

To further investigate the *NSP2* transcript levels in mycorrhizal roots, we analyzed if *NSP2* transcript level shows a correlation to *MtPt4* transcript levels in individual plants. *MtPt4* encodes a phosphate transporter specifically expressed in arbuscule-containing cells [[39]] and can be regarded as a marker for a functional AM symbiosis. We found a clear positive correlation (r = 0.927, P < 0.01) of *NSP2* and *MtPt4* transcript levels (Figure 3). This supports the assumption that *NSP2* is induced in mycorrhizal roots by a mycorrhiza-dependent factor.

### The suppression of functional symbiotic structures by high phosphate fertilization is independent of NSP2

MiR171h transcript levels are increased in plants supplied with high phosphate concentrations. Additionally, at high phosphate conditions mycorrhizal colonization is often decreased as compared to plants grown at phosphate starvation and additionally results in less functional symbiotic structures, namely arbuscules [[28],[40]]. Therefore, is might be assumed that the suppression of mycorrhizal symbiosis at high phosphate conditions is dependent on the miR171h-*NSP2* regulon. To address this question, we inoculated wild type and *nsp2* mutant plants with *R. irregularis* growing in phosphate starvation (20 μM P_i_) or high phosphate (1 mM P_i_) conditions. Transcript levels of MtPT4 as a marker for a functional AM symbiosis were measured at three weeks after inoculation. Mycorrhizal wild-type plants and *nsp2* mutants showed significantly decreased levels of MtPT4 expression when grown at high phosphate fertilization (Additional file 1: Figure S3), which indicates a significant lower frequency of functional symbiotic structures of both lines. Hence, suppression of mycorrhizal colonization by high phosphate fertilization is independent of *NSP2*.

### Induced expression of miR171h at high phosphate conditions is not dependent on CRE1

A recent study of *cis* regulatory elements of the *NSP2* promoter gave evidence that *NSP2*, as well MIR171h, transcription is directly influenced by cytokinin and depends on the cytokinin receptor *CRE1* [[33]]. To analyze whether the induction of miR171h at high phosphate and *NSP2* induction in mycorrhizal roots is dependent on *CRE1*-mediated cytokinin perception, we investigated the relative expression levels of miR171h and *NSP2* in both conditions using *cre1-1* mutant plants and wild-type control plants.

**Figure 4:**
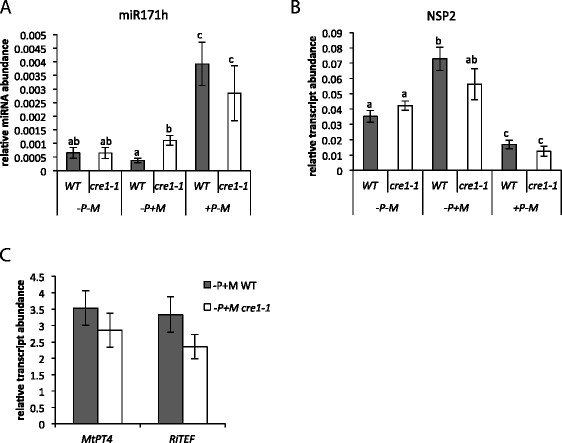
**The** ***cre1-1*** **mutation does not affect phosphate dependent regulation of miR171h as well as** ***NSP2*** **transcript abundance and has no effect on mycorrhizal induced expression of** ***NSP2*** **and arbuscular mycorrhizal marker genes.** The relative expression of mature miR171h **(A)** or *NSP2***(B)** in roots of either WT plants (grey bars) or cre1-1 plants (white bars) determined at different phosphate fertilization and mycorrhizal conditions. -P: 20 μM phosphate, +P: 1 mM phosphate, −M: non-mycorrhizal roots, +M: mycorrhizal roots. **(C)** The relative expression of mycorrhizal the marker genes *MtPt4* and *RiTEF* were determined in root material from the same plants shown in **A** and **B** (−P + M). All plants were harvested 4 wpi. Normalization of the expression data was carried out against a reference gene index (*MtPdf2* and *MtEf1*). Data shown are average values of 3–6 biological replicates. Error bars indicate the standard errors. Different letters indicate statistical different values (P < 0.05). **A** and **B**: two-way ANOVA with Holm-Sidak multiple comparison. **C**: one-way ANOVA).

The *cre1-1* mutation has subtle effect on the *NSP2* transcript abundance compared to wild-type plants in regard to the increased expression in mycorrhizal plants (Figure 4B). On the one hand, only the increase of the *NSP2* transcript abundance in wild-type plants is statistically significant (P ≤ 0.05) compared to *cre1-1* plants, whereas on the other hand there is no statistical significance between wild-type and *cre1-1* plants at mycorrhizal conditions. This might indicate that the transcriptional induction of *NSP2* in mycorrhizal roots is not directly dependent of *CRE1*, however we cannot rule out that *NSP2* expression levels might be indirectly influenced by *CRE1* during arbuscular mycorrhizal symbiosis. We also investigated the effect of *cre1-1* on the AM symbiosis by monitoring the relative transcript levels of the mycorrhizal marker genes *MtPt4* and *RiTEF* [[3],[41]]. Expression level of both genes did not differ between mycorrhizal *cre1-1* and wild type plants (Figure 4C) at 4 weeks post inoculation (wpi), which indicates that *cre1-1* mutants are not impaired in mycorrhizal development. That is also supported by a similar correlation between *NSP2* and *MtPt4* relative transcript levels in *cre1-1* plants compared to the wild type (Additional file 1: Figure S4). In contrast to the nodule symbiosis, where *CRE1* is clearly involved in miR171h and *NSP2* regulation [[33]], these results do not clearly demonstrate that the induction of *NSP2* in mycorrhizal roots is directly mediated by *CRE1*.

Both *cre1-1* and wild type plants showed a significant increase in the amount of miR171h at high P_i_ conditions (1 mM) compared to either non-mycorrhizal or mycorrhizal low P_i_ conditions (20 μM) (Figure 4A). At high phosphate conditions, no significant difference in the relative abundance of miR171h between *cre1-1* and wild type plants could be observed, indicating that the phosphate-induced expression of miR171h is not mediated by *CRE1*.

### Reporter fusions confirm the complex and spatial regulation of NSP2- and miR171h- promoter activities

An increased level of both NSP2 and miR171h in mycorrhizal roots suggests that these transcripts might be spatially separated in roots. We therefore used promoter-reporter fusions to localize the promoter activity of MIR171h and *NSP2* in roots grown at different nutritional and symbiotic conditions. A 1248 bp fragment upstream of the *NSP2* coding sequence and a 900 bp fragment upstream of the miR171h primary transcript were fused to a β-glucuronidase (GUS) reporter gene. Both promoter-reporter constructs were transformed into *M. truncatula* roots by *Agrobacterium rhizogenes*-mediated transformation.

**Figure 5:**
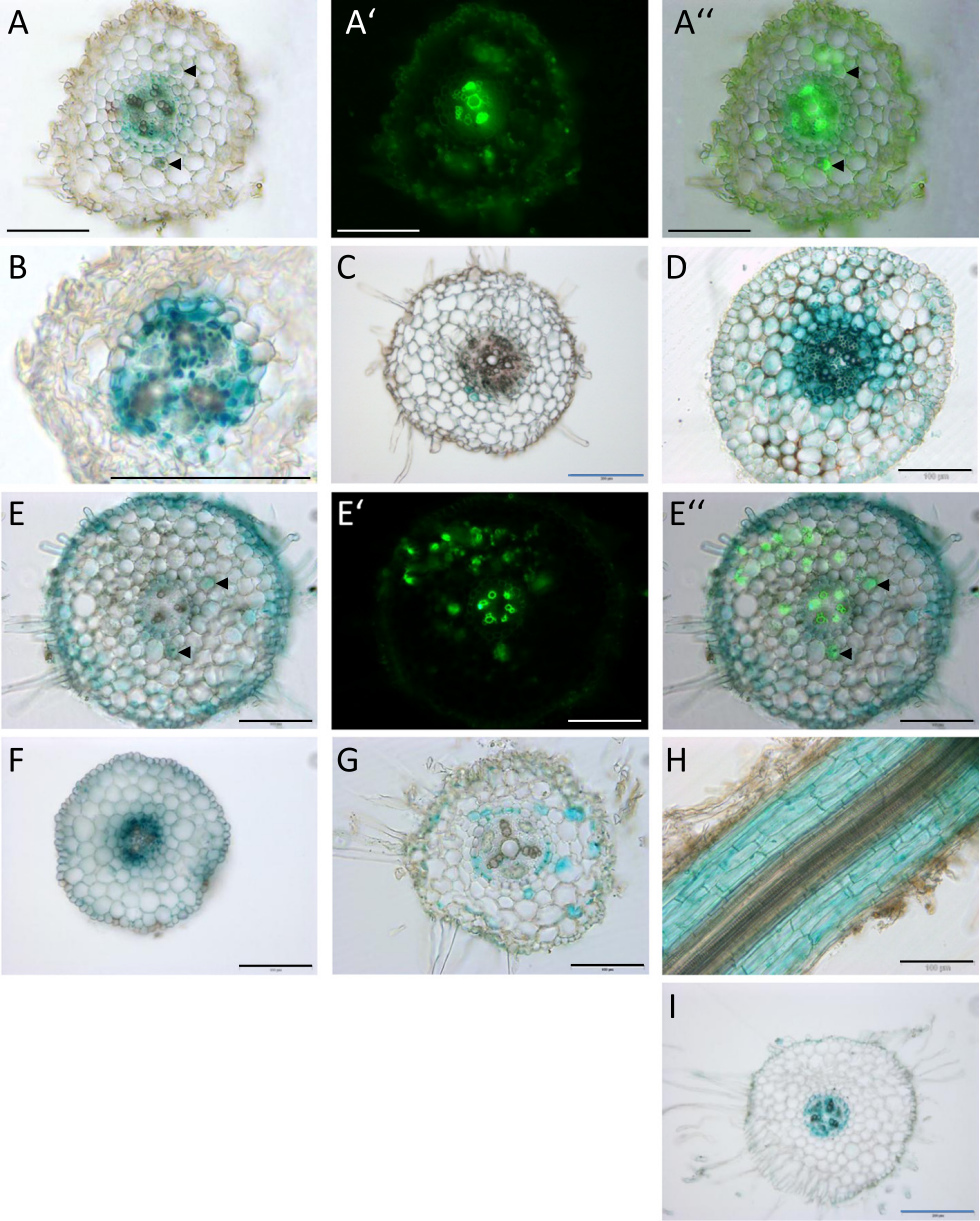
**The** ***NSP2*** **and the miR171h promoter show distinct regulation in response to nutrients and mycorrhizal colonization.** Roots were transformed with promoter-uidA fusions of either the MIR171h **(A-D)** or the *NSP2***(E-I)** promoter. After root transformation, chimeric plants were potted into substrate and grown under different nutritional regimes: phosphate starvation **(B and F)**, phosphate starvation and mycorrhizal colonization **(A and E)**, nitrogen starvation **(C, G, and H)** or full nutrition **(D and I)**. **A**, **A’** and **A”** as well as **E**, **E’** and **E’** represent identical root sections, with **A’/E’** showing WGA Alexafluor 488 fluorescence to visualize *R. irregularis* structures and **A”/E”** showing overlay of bright filed images and WGA Alexafluor 488 fluorescence. Scale bars represent 100 μm (black and white) or 200 μm (blue). Arrowheads point to GUS staining in cortical cells containing arbuscules.

As expected from the transcript accumulation pattern, promoter activity pattern of both MIR171h and *NSP2* showed drastic changes in response to different phosphate and nitrate fertilization treatments (Figure 5). At phosphate starvation, the promoter of MIR171h was mainly active in the central cylinder and the endodermis (Figure 5B). However, after colonization with *R. irregularis*, a weak GUS signal was detectable in distinct, but not in all, arbuscule-containing cells (Figure 5A”). The *NSP2* promoter also showed a strong activity in the central cylinder and endodermis during phosphate starvation. In addition, the *NSP2* promoter showed a weak activity in cortical cells of non-mycorrhizal roots (Figure 5F), whereas in mycorrhizal roots an additional strong activity was observed in the epidermal cell layer, root hairs and in some arbuscule-containing cells of the root (Figure 5E and E”).

This observation might be a reasonable explanation for the above mentioned increased transcript abundance of *NSP2* in mycorrhizal roots (Figure 2 and Additional file 1: Figure S2). During nitrogen starvation no major MIR171h-promoter activity could be observed except in some isolated cortical cells (Figure 5C), which might represent spontaneous promoter activity or staining artifacts. Increased miR171h expression seen during RNS (Figure 2) therefore is not due to the lack of nitrogen fertilization. In contrast to miR171h, the *NSP2* promoter showed a more complex activity pattern in roots of nitrogen-starved plants. Parts of the roots showed *NSP2* promoter activity in apparently random patterns in the cortex cells and endodermis (Figure 5G), whereas in different parts of the same root the promoter activity was observed in the whole cortex (Figure 5H). At full nutrition condition the MIR171h (Figure 5D) and *NSP2* promoter (Figure 5I) showed contrasting localizations, where the MIR171h promoter was active in all root cells with the strongest signals in the central cylinder. In turn, the *NSP2* promoter was not active in the root epidermis and cortex but active in the central cylinder and endodermis. The results of the promoter GUS study in root sections are illustrated in Additional file 1: Figure S5.

**Figure 6:**
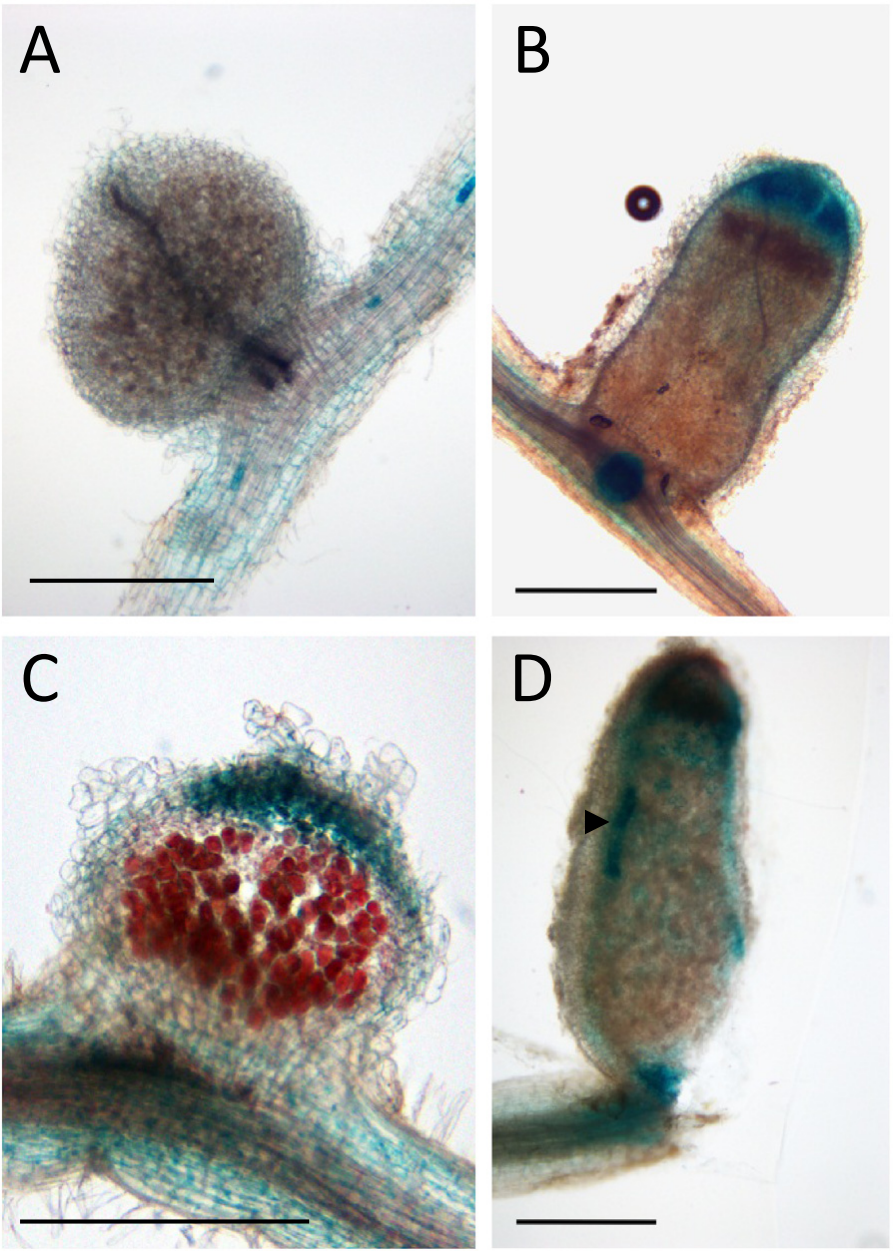
**The** ***NSP2*** **and the MIR171h promoter show distinct expression domains in root nodules.** Roots were transformed with promoter-uidA fusions of either the MIR171h **(A and B)** or the NSP2 **(C and D)** promoter. After root transformation, chimeric plants were potted into substrate and grown under nitrogen starvation additionally colonized with *S. meliloti*. Pictures show either developing nodules **(A and C)** or mature nodules **(B and D)** from the same plants harvested at 5 wpi. Scale bars represent 500 μm. Arrowhead points to a vascular bundle.

Next we analyzed the promoter activity of MIR171h and *NSP2* in root nodules of plants grown at high phosphate, without nitrate and inoculated with *Sinorhizobium meliloti*. Representative young and mature nodules are shown in Figure 6. The MIR171h promoter showed only weak activity in young nodules (Figure 6A) but in mature nodules an increased activity was visible at the nodule tip with the strongest activity matching the meristematic zone and getting gradually weaker in the direction of the subsequent infection and nitrogen fixation zones (Figure 6B). It is worth mentioning that the root cortex cells flanking the nodules showed strong GUS activity, which is in contrast to non-inoculated control roots where no promoter activity was observed in the root cortex (Figure 5C).

The *NSP2* promoter was active in young and mature nodules with the strongest activity in the tip of young nodules and the vascular bundles of mature nodules (Figure 6C and D). Similar to the MIR171h promoter, the *NSP2* promoter was also active in the root cortex in the vicinity of young and mature nodules.

These results demonstrate that the promoter activities of *NSP2* and MIR171h are mainly mutually exclusive and are affected by phosphate as well as nitrate fertilization and by root colonization with *R. irregularis* and *S. meliloti*.

### Mature miR171h accumulates the meristematic zone of root nodules

**Figure 7:**
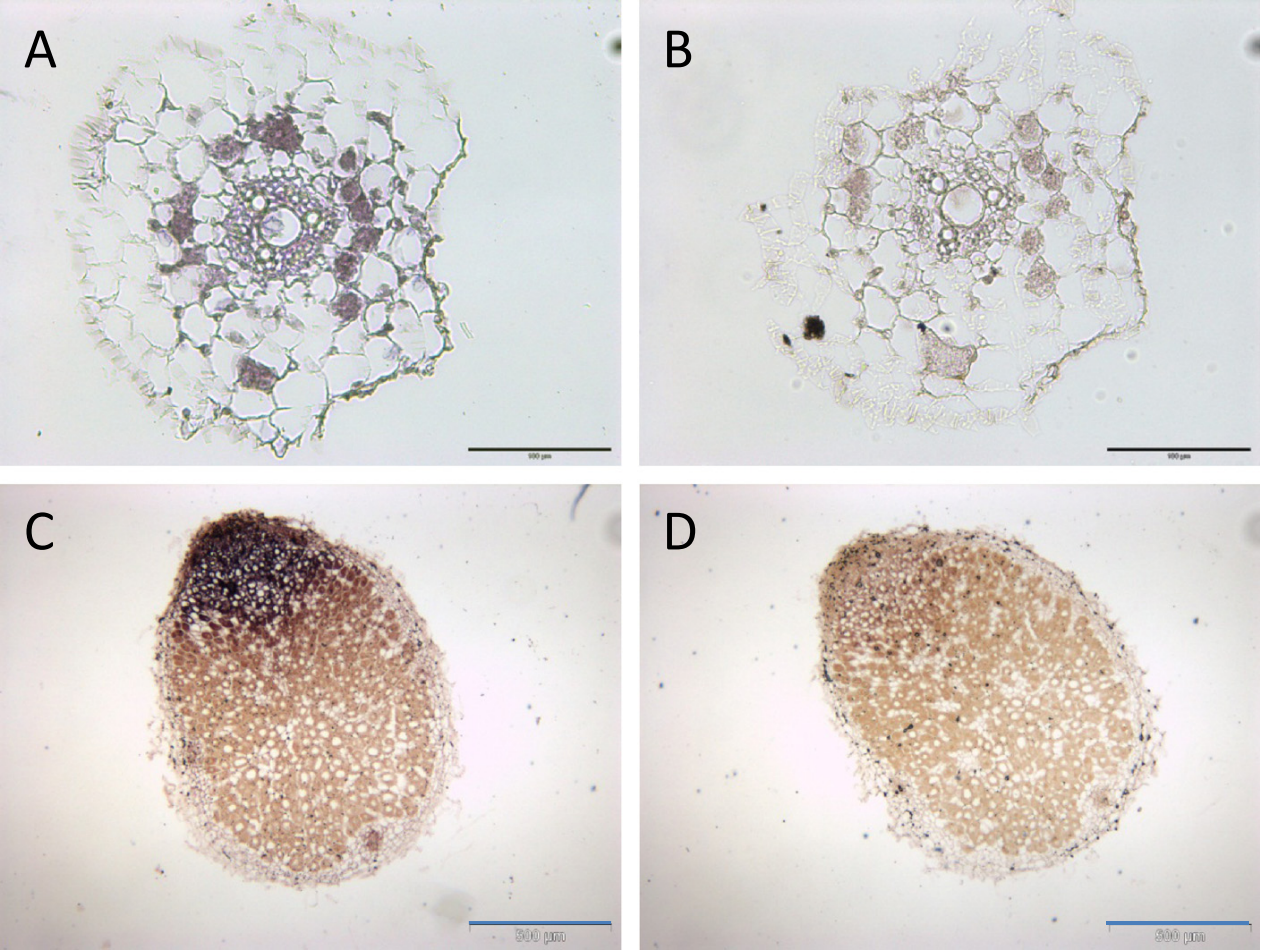
**Localization of mature miR171h accumulation in mycorrhizal roots and root nodules via** ***in-situ*** **hybridization.** Specific miR171h LNA probes **(A and C)** or scramble probes **(B and D)** were used for in situ hybridization to localize mature miR171h molecules in mycorrhizal roots **(A,B)** or root nodules **(C,D)**. Detection was done using NBT/BCIP solution. Scale bars represent 100 μm (black) and 500 μm (blue).

To localize the site of mature miR171h accumulation in mycorrhizal and nodulated roots, we used *in situ* hybridization with a miR171h-specific LNA probe (Figure 7). An LNA probe with a scrambled nucleotide sequence served as a negative control. Consistent with the location of the miR171h promoter activity, we found an accumulation of mature miR171h in the central cylinder of mycorrhizal roots (Figure 7A). We found only weak signals of mature miR171h accumulation in all arbuscule-containing cells, however the MIR171h promoter activity was only observed in distinct arbuscule-containing cells (Figure 5A). Based on the low signal to background ration it is not possible to draw a clear conclusion of its localization but its likely to be in arbuscule containing cells. Localization of the mature miRNA in root nodules was in agreement with the observed promoter activity pattern in mature nodules. Mature miR171h molecules accumulated to the meristematic zone of nodules with the signal intensity gradually decreasing towards the infection zone. No miR171h accumulation could be detected in the nitrogen fixation zone (Figure 7C). These results show that mature miR171h accumulation is consistent with its spatial origin of transcription and point to a protective role of miR171h against target mis-expression in root nodules and probably arbuscule containing cells.

### Over-expression of miR171h leads to a reduced mycorrhizal colonization and reduced nodule numbers

**Figure 8:**
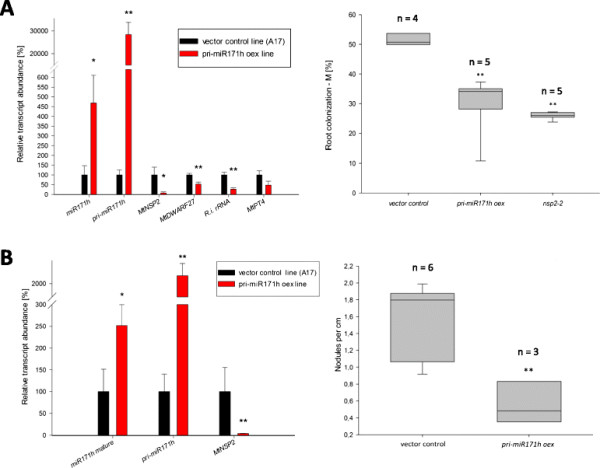
**Ectopic overexpression of MIR171h leads to a strong repression of arbuscular mycorrhizal and root nodule symbiosis.** Roots were transformed either the MIR171h-GFP (red) or the empty vector (black). After root transformation, chimeric plants were potted into substrate and grown under different nutritional and symbiotic regimes: **(A)** 20 μM phosphate inoculated with *R. irregularis*, **(B)** 0 M nitrate and inoculated with *S. meliloti*. Left panes show relative expression values (normalized to *MtPdf2* and *MtEf1*) of selected transcripts and mature miR171h and right panes display box-plots of symbiotic parameters, i.e. **(A)** mycorrhizal root colonization intensity [[42]] and **(B)** number of root nodules per cm of primary root length. Error bars indicate standard errors. Statistical different values between overexpression and vector control plants are indicated by single asterix (P < 0.05) or double asterix (P < 0.01) calculated by student’s t-test. The number of biological replicates is indicated above box plots.

To analyze the biological function of the observed spatial regulation of *NSP2* and miR171h transcription, we ectopically over-expressed miR171h in *M. truncatula* roots. For this purpose, *M. truncatula* roots were transformed with the construct, MIR171h-GFP (Figure 1), already used for the leaf infiltration assay to drive the constitutive expression of pri-miR171h. The empty vector was used as a control. The root-transformed plants were either inoculated with *R. irregularis* or *S. meliloti* to analyze the impact of miR171h over-expression on AMS or RNS, respectively. At 5 weeks post inoculation, over-expression of pri-miR171h led to a very strong accumulation of mature miR171h resulting in a drastic reduction of *NSP2* transcript levels (Figure 8A and B). In addition, we also observed a reduction in *MtDWARF27* transcript abundance, which is a downstream target of *NSP2* and important for converting the strigolactone orobanchol into didehydro-orobanchol [[12]]. Interestingly, we found significantly reduced intraradicular rRNA level of *R. irregularis* but not of *MtPt4* transcript level, which is similar to the unchanged *MtPt4* transcript levels in mycorrhizal roots of *nsp2-2* mutant plants (Additional file 1: Figure S4). The microscopic analysis of the mycorrhizal phenotype [[42]] showed a concomitant reduction in the mycorrhizal intensity to similar levels as the *nsp2-2* mutant line (Figure 8A), but no significant reduction of the arbuscule abundance was observed in the pri-miR171h overexpressing plants (Additional file 1: Figure S6). This implies that plant roots which over-accumulated mature miR171h were less colonized by the mycorrhizal fungus whereas arbuscule development in colonized root areas was not impaired. Additionally, we also found significantly reduced nodule numbers in roots over-expressing mature miR171h compared to the vector controls (Figure 8B).

In summary, ectopic overexpression of the primary miR171h transcript in roots of *M. truncatula* is leading to a phenotype analogous to the *nsp2-2* mutant phenotype during mycorrhizal and nodule symbiosis.

## Discussion

One of the recently identified miRNAs, which specifically target genes essential for root endosymbiosis is mtr-miR171h [[30]].

MiR171h has later been predicted to be produced from an 811 bp long primary transcript encoding two miR171h loci on a single arm of its fold-back structure [[31]]. In this work, we provided experimental proof that miR171h is functionally expressed from an at least 811 bp long primary transcript. Cleavage of *NSP2* transcripts by miR171h has been verified by RACE-based methods [[30],[32],[36]]. However, so far functional analysis of miR171h has been carried out with the primarily identified partial precursor encoding only a single miRNA duplex [[32]]. By using a specific miRNA-sensor construct in a *N. benthamiana* leaf infiltration assay we could confirm *in-vivo* that the long double duplex-containing MIR171h primary transcript exhibits biological activity. This suggests that the long primary transcript described by Branscheid *et al.* [[28]] resembles the endogenous source of mature miR171h in *M. truncatula*.

The target of miR171h, *NSP2*, is a GRAS-type transcription factor, which is essential for the formation of root nodule symbiosis [[13],[43],[44]], strigolactone biosynthesis [[12]] and is involved in the Myc-factor signaling pathway [[7]]. It has been recently shown that miR171h overexpression leads to a reduction of mycorrhizal root colonization [[32]] and it has been proposed to act specifically in arbuscular mycorrhizal symbiosis. Expression data of miR171h implies that this miRNA is induced by full nutrition conditions [[30]] and we hypothesized that miR171h is regulated by the phosphate status of the plant. Data from Arabidopsis and Rapeseed did not imply that the miR171 family is regulated by phosphate and nitrogen availability [[45]]. However, the data from the current study give strong evidence that the expression of miR171h is mainly induced by high phosphate availability (Figure 2). It is possible that the phosphate dependent regulation of a miR171 family member is dependent of the ability to undergo root endosymbiosis, because the miR171h isoform is only present in AMS capable plants [[27],[32]]. Whether additional miR171 family members are phosphate responsive in these plant species is not known. Interestingly, the promoter GUS reporter assays suggest that the expression of miR171h is not merely regulated by phosphate *per se* but rather by a combination of phosphate and nitrogen levels, because the activity of the miR171h promoter is absent at high phosphate and low nitrogen conditions compared to full nutrition conditions (Figure 5C and D).

Recently, it has been shown that miR171h expression is induced by cytokinin treatment and dependent on the *CRE1* pathway [[33],[46]] i.e. that the loss of *CRE1* leads to loss of miR171h induction by cytokinins. Because it is known that P_i_ and nitrogen fertilization increases cytokinin levels of plants [[47],[48]] and elevated levels are also found in shoots and roots of mycorrhizal plants [reviewed in 35]. Based on this knowledge we hypothesized that the expression levels of miR171h at high phosphate conditions might be dependent on *CRE1* as a direct effect of induced cytokinin levels in these plants. We used the *cre1-1* mutant plant line to address this question. The presented results indicate that phosphate dependent regulation of miR171h and its modest induction in mycorrhizal roots is not dependent on *CRE1*; the latter assumption is in agreement with the fact that the *cre1-1* mutation has no statistically significant effect on the mycorrhizal marker gene expression and thus mycorrhizal colonization (Figure 4C). Taken together we conclude that the miR171h expression is regulated by the phosphate status of the plant in a nitrogen dependent manner controlled by an unknown genetic factor, in addition to the previously demonstrated inducing effect of myc-LCOs [[32]].

Interestingly, we found elevated transcript levels of *NSP2* in mycorrhizal roots (Figure 2 and Additional file 1: Figure S2) between two and six weeks post inoculation. Similar to miR171h, the expression of *NSP2* is induced upon cytokinin treatment in a *CRE1*-dependent manner during high phosphate and low nitrogen conditions [[33]]. However, the involvement of *CRE1* in the induction of *NSP2* transcript abundance in mycorrhizal roots during low phosphate and high nitrogen conditions was not as clear as with the cytokinin treatment mentioned above (Figure 4B). Therefore, we assume that this up-regulation is mediated by an unknown factor and does not involve cytokinin perception *per se.* We cannot rule out a subtle influence *CRE1* on *NSP2* expression during arbuscular mycorrhizal symbiosis, though mycorrhizal colonization itself is clearly not dependent on the *CRE1*-pathway. The observed increase in *NSP2* transcript levels is most likely the result of the observed additional promoter activity in the epidermal cell layer of mycorrhizal roots compared to non-mycorrhizal roots (Figure 5E and F) and which lack MIR171h promoter activities (Figure 5A). We cannot rule out the possibility that this phenomenon is due to the lack of proper loading of Argonaute proteins, the major component of the RNA induced silencing complex (RISC), with mature miR171h, but due to a lack of antibodies against *M. truncatula* AGO1 homologues, this assumption awaits to be tested. Additionally, *NSP2* might be preferably repressed by a translational rather than post-transcriptional repression [[19]], which would leave the steady-state transcript abundance unchanged. However, owing to the mutually exclusive expression pattern of the miR171h-*NSP2* target pair during mycorrhizal symbiosis as discussed below, we favor the aforementioned explanation. Yet, our observation of an induced *NSP2* expression during mycorrhizal conditions is in contrast to the previously published results of Lauressergues *et al*. [[32]] who observed a slight decrease in *NSP2* transcript levels in mycorrhizal roots. The exact cause for this discrepancy is not known, but we could show that our results are consistent over 3 independent experiments: (I) by qRT-PCR measurement of *NSP2* transcript levels based on material 3 weeks post inoculation as shown in Figure 2, (II) by qRT-PCR measurement based on material from a time-course over 6 weeks as presented in Additional file 1: Figure S2, and (III) by qRT-PCR measurement based on the parental line of the *cre1-1* mutant, harboring a WT gene, 4 weeks post inoculation as shown in Figure 4. Moreover a correlation of single data points derived from individual plants based on the measurement shown in Figure 4 shows a positive correlation between *MtPt4* (as a measure of arbuscule abundance) and *NSP2*. Additionally, we reinvestigated publically available microarray datasets, which are deposited in the Medicago Gene Expression Atlas v3 (MtGEA; http://mtgea.noble.org) using the probe Mtr.44789.1.S1_at, which corresponds to *NSP2*. Although the data of Gomez *et al*. [[49]] shows only a slight increase of NSP2 in mycorrhizal conditions at best, but no reduction as well, the data of Hogekamp *et al.* [[50]] shows a strong increase in the signal of the probe in response to 6 weeks of colonization by *R. irregularis* or *Glomus mosseae* and a strong decrease in response to 2 mM Pi treatment compared to 20 μM treated control conditions. Analysis of the supplementary data from Hogekamp *et al.* [[50]] in more detail revealed that the increase of *NSP2* expression in *R. irregularis* treated roots has a log2 fold change of 0.7 (p-value = 0.02) and in *G. mosseae* 0.84 (p-value = 0.008), which represents a roughly 1.7× times induction of *NSP2* expression during AM symbiosis, which is not only in agreement with our data and strongly supports our results, but also indicates that this response is conserved in symbiotic interactions with *G. mosseae*. As the slight induction in the data of Gomez *et al.* [[49]] is not significant and Lauressergues *et al.* [[32]] observed a reduction in *NSP2* levels upon *R. irregularis* colonization it is clear that there is at least one other unknown factor involved in the regulation of the miR171h-NSP2 target pair. These might involve specific regulation by additional miR171h target genes [[30],[32]] not investigated in this study, which might be preferably silenced by miR171h instead of *NSP2* at the conditions tested by us. Future detailed analysis of their spatial expression pattern from promoter to the protein level, the latter also accounts for *NSP2*, might give insight into this conundrum.

When comparing the spatial distribution of the MIR171h- and *NSP2*-promoter, it becomes evident that they predominantly show a mutually exclusive expression pattern, especially at full nutrition (cortex and root epidermis) and mycorrhizal conditions (root epidermis). Such a pattern has also been described for the miR395-SULTR2;1 miRNA target pair [[51]] and most likely functions to prevent expression of transcripts resulting from spontaneous promoter activity in specific tissues and cell types, a mechanism called promoter dampening [[18]]. That this model applies to the miR171h-*NSP2* target pair is in agreement with the results from the ectopic mis-expression of miR171h by 35S driven expression which leads to a drastic reduction in *NSP2* transcript levels and mycorrhizal colonization (this study and [[32]]) as well as with the 35S driven ectopic mis-expression of a miR171h-resistant NSP2 version leading to over-colonization of the root system and root apices, whereas ectopic mis-expression of the wild type *NSP2* transcript, which can be cleaved by miR171h, does not [[32]]. This mutually exclusive expression pattern is especially pronounced at full nutrition conditions where the miR171h is strongly expressed in all root tissues, whereas the *NSP2* promoter is completely inactive with the exception of the central cylinder. This assumes that the plant needs to comprehensively ensure a tight *NSP2* expression control in roots of nutrient replete plants to avoid the formation of root nodule and AM symbiosis, especially at full nutrition supply. Even though we cannot exclude the possibility that miR171h induction by high phosphate fertilization has an impact on mycorrhizal repression by restricting *NSP2* expression, we rule out that this effect is solely dependent on miR171h, because *nsp2-2* plants still respond to high phosphate treatment with repression of mycorrhizal colonization (Additional file 1: Figure S4) even though the *NSP2* dependent regulation is genetically knocked out, indicating that other regulatory circuits act epistatically to the miR171h-*NSP2* regulatory circuit. Additionally, a recent important discovery is that NSP2 interacts with another GRAS-type transcription factor RAM1, which is essential for mycorrhizal colonization and hyphopodia formation by controlling the expression of *RAM2* [[15],[16]]. RAM2, a glycerol-3-phosphate acyl transferase, catalyzes an important step in cutin monomer production, which seems to be an important molecule for promoting hyphopodia attachment to the plant root, and thus can also be hijacked by filamentous pathogenic fungi, e.g. *Phytophthora palmivora* [[16]]. Accordingly, it might be assumed that the mutually exclusive expression pattern of miR171h has been evolved to avoid hijacking of the *NSP2* signaling pathway by pathogens, creating only a narrow conditional window to allow the formation of beneficial symbiotic interactions, presumably relying on a previous signal from a beneficial symbiotic partner. We therefore conclude that miR171h acts as a promoter dampener, which protects specific cell-types of the root at distinct nutritional and symbiotic conditions from *NSP2* mis-expression in a mutually exclusive expression pattern, whereas the overall transcript abundance of *NSP2* in wild-type *Medicago truncatula* roots colonized by arbuscular mycorrhizal fungi is mainly determined by its promoter activity rather than direct cleavage by miR171h.

Other miRNA regulatory circuits often seen by miRNA-target pairs beside promoter dampening are spatial restriction and temporal regulation [[18]]. An exemplary spatial restriction type important in regulating root nodule development has been documented for miR169 in *M. truncatula* [[25]]. There, miR169 restricts the expression of its target *MtHAP2-1* to the meristematic zone of the root nodule, to allow correct maturation of the root nodule. The data of our study suggests that spatial restriction seems to be a predominant regulatory mechanism of miR171h during root nodule development, in contrast to the segregated expression pattern discussed above. In root nodules the MIR171h and *NSP2* promoter show an overlapping spatial distribution (Figure 6), with the *NSP2* promoter being active in the whole nodule. MIR171h transcription and accumulation is restricted to the meristematic zone, which was confirmed by *in-situ* hybridization (Figure 7). Ectopic over-expression of the long miR171h primary transcript led to a significant reduction in the number of root nodules (Figure 8) and therefore could demonstrate that miR171h over-expression represses root nodule symbiosis, additional to its described capability to restrict mycorrhizal root colonization [[32]]. Furthermore, a recent study from De Luis *et al.* [[36]] in *L. japonicus* identified a homologue of miR171h, lja-miR171c, in determinate root nodules, which similarly targets *LjNSP2*. Its expression in nodules is dependent on the presence of *Mesorhizobium loti*, which indicates that the regulation of miR171h expression has a higher level of complexity than previously thought. Previous attempts to overexpress miR171h and miR171c in *M. truncatula* and *L. japonicus* failed, which could be explained by the use of the 35S promoter, which seems to be diminished in arbuscule containing cells [[52]] and root nodules [[53],[54]] and thus might lead to a variation of the results. Our demonstrated phenotype might be explained by a dosage dependent effect of the miR171h overexpression, because the primary transcript used in this study encodes two miR171h duplexes [[31]] and thus the maximum abundance has likely been doubled, balancing the lower 35S activity in root nodules. Taken together, our data provides profound evidence that miR171h can directly influence the root nodule symbiosis and we hypothesize that miR171h protects the root of the plant from *NSP2* mis-expression in the meristematic zone of indeterminate nodules and therefore prevents bacterial invasion of this tissue, analogous to the protection of the root tip from colonization with mycorrhizal fungi [[32]].

## Conclusion

Our data revealed that the spatio-temporal expression of miR171h and *NSP2* is tightly linked to the nutritional status of the plant and reflecting the different physiological conditions at which both types of endosymbiosis are favored by a host legume plant. Together with the results from the overexpression analysis, our data points to an important function of miR171h to integrate the nutrient homeostasis in order to safeguard the expression of *NSP2* during both, arbuscular mycorrhizal and root nodule symbiosis.

## Methods

### Plant materials and growth conditions

Seeds of *Medicago truncatula* cv. Jemalong (A17), *nsp2-2* and *cre1-1* mutant plants were germinated as described in [[28]]. Seedlings were grown in a quartz sand (0.6 - 1.2 mm), vermiculite and expanded clay substrate (7:1:1 [v/v]) with 16 h light (25°C) and were twice a week fertilized, with half a strengths Hoagland solution containing 20 μM Pi. For high Pi conditions the solution contained 1 mM Pi and for experiments under N limitation Ca(NO_3_)_2_ and KNO_3_ were substituted by CaCl_2_ and KCl, respectively. For mycorrhizal colonization the seedlings were inoculated with *R. irregularis* by mixing the substrate with 10% (v/v) substrate from *Allium porrum* plants, which were used as host plants to propagate *R. irregularis* for at least 2 months. Plants used for analysis were harvested at time points indicated in the results section and representative parts of the roots were stained with WGA-Alexa Fluor 488 (Invitrogen) to estimate mycorrhizal colonization. Remaining roots were immediately frozen in liquid nitrogen and immediately used for total RNA extraction or stored at −80°C.

### Molecular cloning

The 811 bp fragment of pri-miR171h located at the genomic position 31,405,065..31,404,255 of chromosome 3 (Mt3.5) [[31]] was cloned into the *Cauliflower mosaic* virus 35S (short 35S) driven binary vector pK7WG2D [[37]]. The resulting construct was named MIR171h-GFP.

The miR171h binding site of MtNSP2 and a miR171h non-cleavable sequence (scramble) were obtained by gene synthesis by Eurofins® (MWG® Operon) and cloned into the 35S driven binary vector pGWB455 [[55]] used for N-terminal mRFP protein fusions, resulting in the constructs MBS-NSP2 or MBS-mut, respectively. Both sequences contained stop codons in frame with mRFP.

Promoter regions of mtr-miR171h and MtNSP2, 900 bp as well as 1248 bp upstream of the start codon, respectively, were amplified from wild type (*Medicago truncatula* cv. Jemalong) genomic DNA and cloned into the binary vector pKGWFS7 containing GFP:GUS fusion protein driven by the inserted vector.

### Root transformation and leaf infiltration assays

Prior to root transformation seeds were surface sterilized (12 min conc. H_2_SO_4_, 5 min 6% NaClO) and germinated (2 days dark, 4°C, 2 days dark, 25°C) on 0.8% [w/v] water agar.

Roots of *Medicago* seedlings were transformed with *Agrobacterium rhizogenes* (ARqua1) as published by [[56]]. In short, roots were cut with a sterile razor blade and wounded site was dipped on a two day old *A. rhizogenes* culture, transferred to 0.8% [w/v] water agar in the dark for two days before transferring to selective Fahraeus medium containing 25 μg/ml of kanamycin and cultivated for 3 weeks in the phytotron (12 h/12 h light/dark cycle).

Tobacco leaf infiltration essay was conducted using 4 weeks old *Nicotiana benthamiana* plants prior to the flowering phase. All experiments were performed twice in three independent plants. Tobacco plants were saturated with water 2–4 h before infiltration. *Agrobacterium tumefaciens* (GV2260) containing the appropriate constructs was inoculated in 20 ml YEB medium containing the appropriate antibiotics and grown for two days at 28°C and 250 rpm in the dark. Cultures were pelleted in 50 ml falcon tubes by centrifugation for 15 min at 4000 rpm (Eppendorf, 5804), re-suspended in ice cold AS medium containing 10 mM MES, 10 mM MgCl_2_, 150 μM Acetosyringon pH 5.6 and diluted to an OD_600_ of 0.8 to 1 with AS medium and further incubated for 3 h at RT under soft shaking (~50 rpm). Infiltration was carried out as co-infiltration essay were Agrobacteria cultures containing different constructs were mixed 1:1 prior to infiltration. For single construct infiltration event, Agrobacteria, containing the respective construct, were mixed 1:1 with empty Agrobacteria. Each leaf was infiltrated with approximately 500 μl into the abaxial side of the tobacco leaf according to [[57]]. Plants were grown under moderate light conditions for another 3 days before checked for fluorescence of the reporter proteins.

### Inoculation with *Sinorhizobium meliloti*

Inoculation of *M. truncatula* roots with *S. meliloti* strain 1021 was carried out as described in [[30]].

### RNA extraction

The RNA was extracted using the miRVANA miRNA isolation kit (Ambion) with a previous Plant Isolation Aid step (Ambion) according to the manufacturer’s protocol.

### RT- and qRT-PCR analysis

Quantitative RT-PCR analysis was carried out as described previously [[30]]. In brief, qRT-PCR analysis was performed in a 10 μl reaction volume. cDNA was diluted 1:10 and 1 μl was used for the reaction. The mixture contained 4 μl of 0.5 μM primer-pair mix and 5 μl Maxima™ SYBR Green/ROX qPCR Master Mix (Fermentas). The qRT-PCR machine, ABI Prism 7900 HT (Applied Biosystems), was used with the following reaction set up: first step 95°C for 10 min, 40 cycles 95°C for 15 sec and 60°C for 60 sec, alternately. Finally, a dissociation stage (95°C for 15 sec, 60°C for 15 sec heating with ramp rate of 2% up to 95°C for 15 sec) for melting curve analysis was included. Data was collected and analyzed using SDS 2.4 (Applied Biosystems) and SigmaPlot v.12 (Systat). The threshold for Ct-values was set to 0.2 with an automatic baseline correction for all experiments. The averages of the Ct-values of *MtEF1-α*, *MtPDF2* and *MtGAPDH* were used to calculate the housekeeping gene-index (HK-index) for normalization of transcript abundance. Functional symbiotic structures where determined by measuring the *MtPt4* transcript abundance [[3]] and the relative colonization level was determined by measuring the *RiTEF* transcript abundance [[41]].

### *In situ* hybridization with Digoxygenin (DIG) -labeled LNA probes

The *In situ* hybridization was carried out as described previously [[30]]. Before embedding the roots and nodules into paraffin they were fixed under vacuum using fixative (4% paraformaldehyde in PBS, pH7). LNA probes against mtr-miR171h were custom designed by Exiqon.

### GUS staining and root sectioning

β-glucuronidase (GUS) activity was used to assay miR171h and *NSP2* promoter activity as described in [[58],[59]]. Plants were grown under different conditions, as described earlier and harvest after 4 wpi. Shoots were removed and roots were incubated in GUS staining buffer containing 100 mM NaPO_4_ 1 mM K_3_[Fe(CN)_6_], 1 mM K_4_[Fe(CN)_6_], 10 mM EDTA and 5-bromo-4-chloro-3-indolyl glucuronide, pH 7 at 37°C for different time scales, as indicated. Roots were embedded in 4% [w/v] agarose and 40 μm thick cross and longitudinal sections were obtained using the Leica VT 1000S vibratome (Bensheim, Germany).

### Microscopic analysis

Images of sections were generated using an Olympus BX41 microscope with the cellP software (Olympus, Hamburg, Germany) using different zoom objectives as indicated.

Images of tobacco leafs were performed using the Stereo-Fluorescence Microscope MZ 16FA (Leica) with a PLANATO 1× objective (DC-DFC 300FX 1.3 MP, M.115×). Fluorescence proteins were excited with a mercury metal halide bulb laser, filtered with GFP3 filter system (exciting: 470/40 nm, barrier: 525/50 nm) for GFP fluorescence and a DsRED filter system (exciting: 545/30 nm, barrier: 620/60 nm) for mRFP fluorescence. Bright-field images were taken with identical settings except no laser-beam or filters were used. Exposure time for fluorescence pictures were 9 seconds with gain 5 and zoom factor 11.4×. Filters were successively shifted without changing settings. Image data were processed with the Leica LAS-AF Version 2.8.1.

### Determination of mycorrhizal parameters

Mycorrhizal colonization parameters of *Medicago* roots were determined by stained with Alexa Fluor® 488 Dye according to [[60]]. Roots were cleared with 10% [w/v] KOH for 5 min at 90°C. Roots were incubated at room temperature for at least 24 h in Alexa Fluor staining solution, containing 137 mM NaCl, 2.7 mM KCl, 12 mM Na_2_HPO_4_/KH_2_PO_4_ and 0.01% [w/v] Alexa Fluor® 488 conjugate of wheat germ agglutinin (Invitrogen), and then washed five times prior mounting on microscopy slides. Mycorrhizal parameters defined by [[42]] are F%: Mycorrhizal frequency, M%: Mycorrhizal intensity per root, m%: Mycorrhizal intensity per root fragment A%: Arbuscule intensity per root, a% Arbuscule intensity per root fragment.

### Protein extraction and western blotting

Protein extraction and western blotting was carried out as described previously [[61]]. Protein concentration was determined using Bradford kit (Biorad) with provided γ-globulin standard following the manufacture’s protocol.

Proteins were diluted to a final concentration of 1.5 μg/μl with homogenization buffer (100 mM HEPES, 10% [v/v] Glycerol, 5 mM dithiothritol, 1 tablet cOmplete™ ULTRA Tablets (Roche) per 10 ml buffer pH 7.5). 10 μl of protein solution was mixed with 10 μl of 2 × SDS sample buffer (125 mM Tris, 20% [v/v] glycerol, 4% [w/v] SDS, 0.01% [w/v] bromophenol blue, 40 μM DTE) and incubated for 5 min at 95°C. Samples were centrifuged at 14000 g for 1 min (4°C) and then resolved on a 1 mm 12% SDS polyacrylamide gel. The semi-dry blot was assembled as described in the Immobilon®-P Transfer Membrane (Millipore™) user guide. The blot was disassembled and the membrane was further used for immunostaining. Detection of GFP and mRFP was carried out using 1:2500 and 1:1000 dilutions for anti-GFP (rabbit, GenScript) and anti-mRFP (rabbit, Abcam®), respectively. As a loading control, a 1:10,000 diluted anti RubisCO antibody (rabbit, Agrisera™) was added to each of the antibody solutions. The secondary anti-rabbit-alkaline-phosphatase (Abcam®) antibody was diluted 1:5000. For visualization a ready-to-use NBT/BCIP solution (Roche®) was used. The membrane was dried and scanned with the Epson scanner Perfection 4870 (Epson Scan 3.0 Software). RubisCO, mRFP and GFP were identified by comparison to a pre-stained protein size marker (Thermo Scientific).

## Competing interest

The authors declare that they have no competing interests.

## Authors’ contributions

VH, EAD, NG and AM performed biological experiments. EAD and FK designed and initiated the research. All authors analyzed the data. EAD and FK wrote the manuscript. All authors read and approved the final manuscript.

## Additional file

## Supplementary Material

Additional file 1: **Includes the following supporting figures:Figure S1.***In vivo* confirmation of *NSP2* gene silencing by miR171h using miR171h overexpression and mRFP sensor constructs. Co-infiltra2on of miR171h overexpression and mRFP sensor constructs. **Figure S2.** Expression profile of mycorrhizal marker genes, pri-miR171hand *NSP2* during arbuscular mycorrhizal colonization of *M. truncatula* roots. **Figure S3.** Mycorrhizal colonization can be repressed by high phosphate in *nsp*2 mutants. **Figure S4.** The *cre1-1* plant line shows a similar correlation between the relative *NSP2* and *MtPt4* transcript abundance like wild type plants. **Figure S5.** Cartoon representation summarizing the localization of the *NSP2* and MIR171h promoter. **Figure S6.** Mycorrhizal parameters from root transformed wild type and miR171h overexpression plants compared to *nsp2-2* plants. Click here for file
