# Supplementary material for: MiR171h restricts root symbioses and shows like its target NSP2 a complex transcriptional regulation in Medicago truncatula
Source: BMC Plant Biol. 2014 Jul 23;14:199. doi: 10.1186/s12870-014-0199-1 (PMC4115173; doi:10.1186/s12870-014-0199-1)
Supplement: Additional file 1: — Includes the following supporting figures:Figure S1.In vivo confirmation of NSP2 gene silencing by miR171h using miR171h overexpression and mRFP sensor constructs. Co-infiltra2on of miR171h overexpression and mRFP sensor constructs. Figure S2. Expression profile of mycorrhizal marker genes, pri-miR171hand NSP2 during arbuscular mycorrhizal colonization of M. truncatula roots. Figure S3. Mycorrhizal colonization can be repressed by high phosphate in nsp2 mutants. Figure S4. The cre1-1 plant line shows a similar correlation between the relative NSP2 and MtPt4 transcript abundance like wild type plants. Figure S5. Cartoon representation summarizing the localization of the NSP2 and MIR171h promoter. Figure S6. Mycorrhizal parameters from root transformed wild type and miR171h overexpression plants compared to nsp2-2 plants. [file s12870-014-0199-1-S1.pdf]

Figure S1

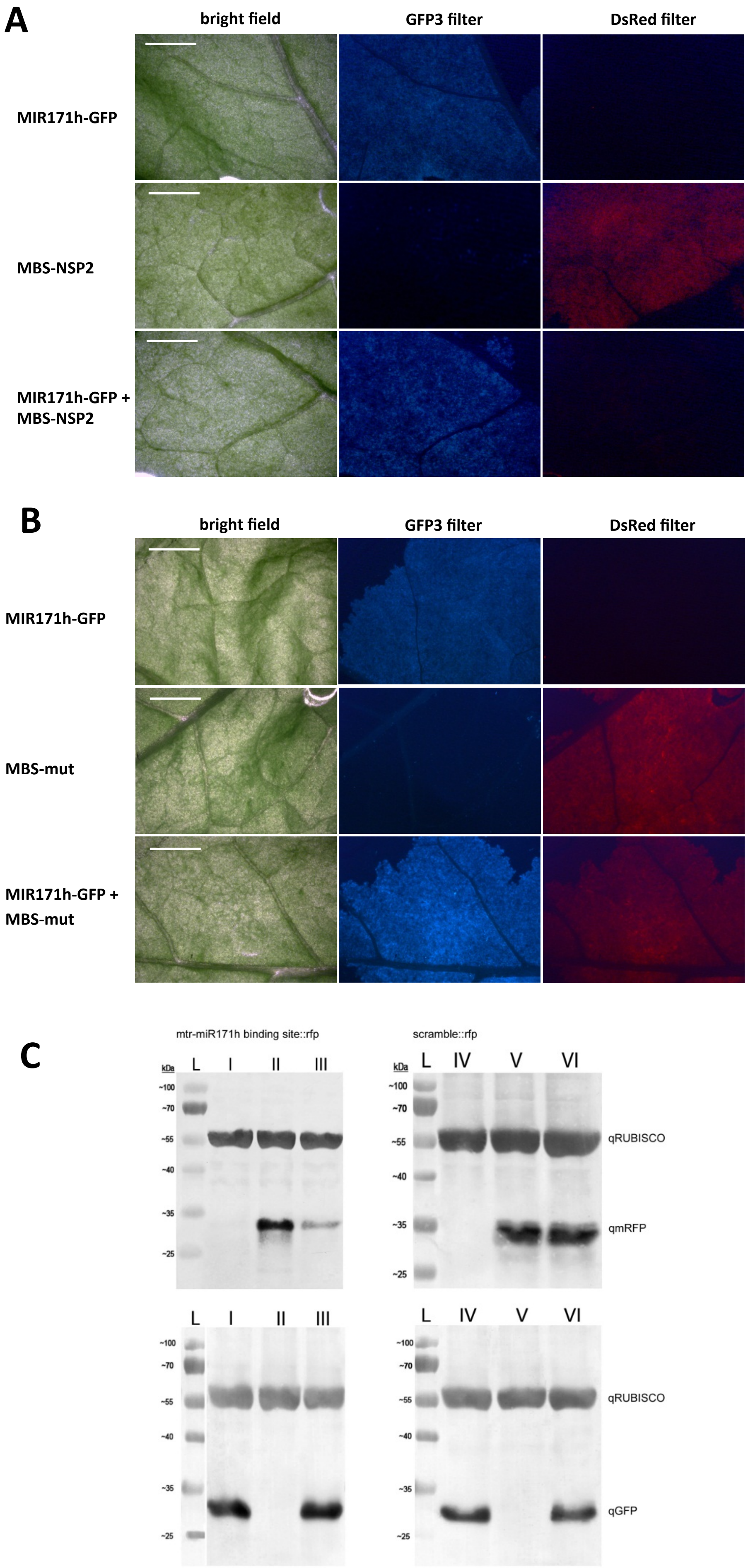
A
bright field
GFP3 filter
DsRed filter
MIR171h-GFP
MBS-NSP2
MIR171h-GFP +
MBS-NSP2
B
bright field
GFP3 filter
DsRed filter
MIR171h-GFP
MBS-mut
MIR171h-GFP +
MBS-mut
C
mtr-miR171h binding site::rfp
scramble::rfp
kDa
L
I
II
III
IV
V
VI
~100
~70
~55
~40
~35
~25
qRUBISCO
qmRFP
qGFP

Figure S1

In vivo **confirmation of** ***NSP2*** **gene silencing by miR171h using miR171h overexpression and mRFP sensor constructs.**

Co-infiltration of miR171h overexpression and mRFP sensor constructs. *Nicotiana benthamiana* leaves were infiltrated with single MIR171h-GFP and either MBS-NSP2 (A) or MBS-mut (B) (upper and middle panels). For each sensor construct, co-infiltration experiments with the MIR171h-GFP construct were carried out (lower panels). Bright filed images, GFP3 fluorescence and mRFP fluorescence are shown. Scale bar: 5 mm.

(C) Full Western blot membranes seen in Figure 1. Proteins were extracted from leaves infiltrated with MIR171h-GFP, MBS-NSP2 or MBS-mut and co-infiltration of both constructs. The upper two blots were incubated with an α-mRFP antibody; the lower two blots were incubated with an α-GFP antibody, serving as a control of the MIR171h-GFP transformation efficiency. On both blots, RuBisCO proteins were detected to demonstrate equal loading of the protein samples.

Figure S2

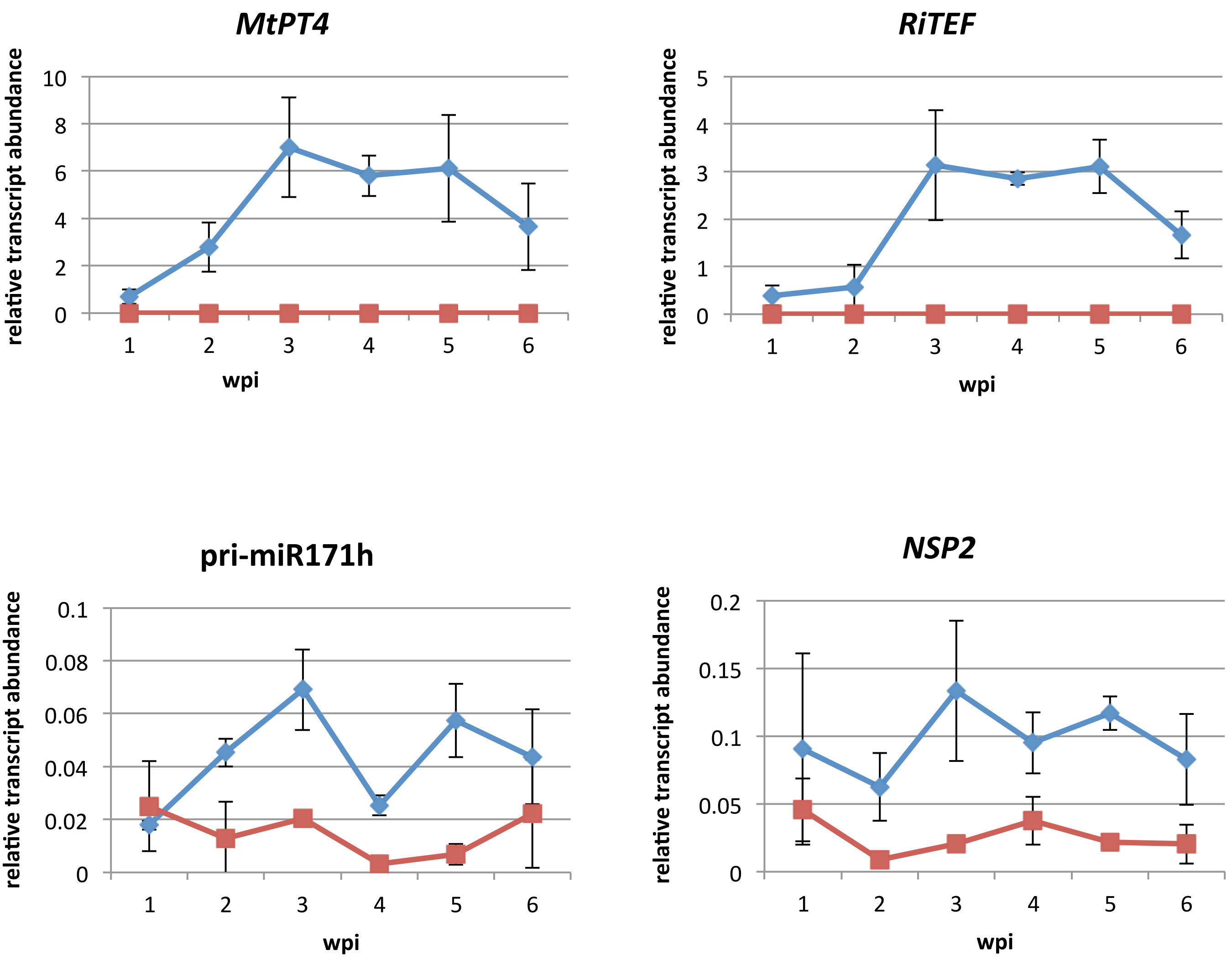

Figure S2

Expression profile of mycorrhizal marker genes, pri-miR171h and *NSP2* during arbuscular mycorrhizal colonization of *M. truncatula* roots.

Quantitative real-time PCR measurements on cDNA derived from *M. truncatula* root tissues of a time-course of arbuscular mycorrhizal colonization (blue) during 1 to 6 weeks post inoculation (wpi) compared to a mock-inoculated control (red). The plants were fertilized with 20 µM Pi. Normalization of the expression data was carried out against a reference gene index (*MtPdf*2 and *MtEf*1). *MtPt4* is a molecular marker for functional symbiotic structures [3] and *RiTEF* is a molecular marker for mycorrhizal colonization [39]. Data shown are average values of 4 biological replicates, except for 3 wpi which represents an average of 3 biological replicates. Error bars indicate the standard errors.

Figure S3

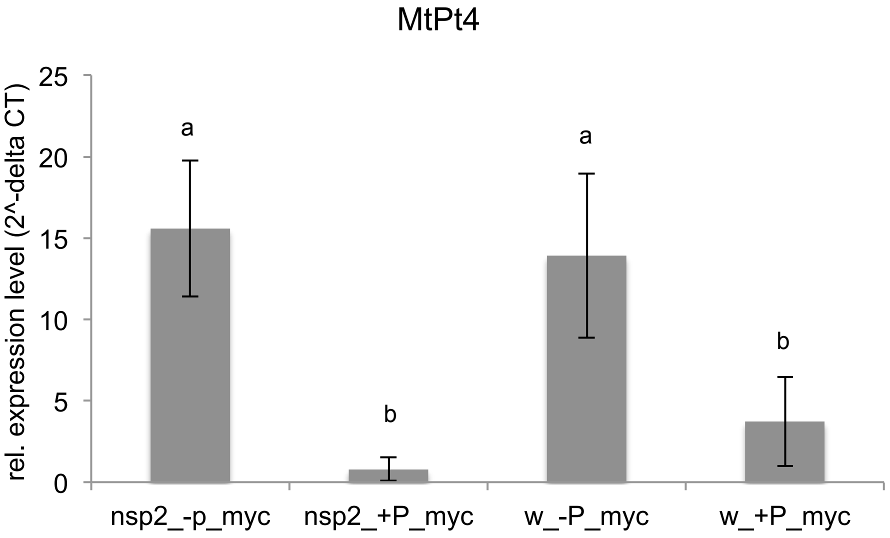

Figure S3

Mycorrhizal colonization can be repressed by high phosphate in *nsp*2 mutants.

A: *MtPt4* transcript levels were measured in mycorrhizal roots grown at either 20 µM phosphate (-p) or 1 mM phosphate (+p) by quantitative RT-PCR analysis. Mycorrhizal wildtype plants (w) or *nsp2-2* mutants (nsp2) were inoculated with *Rhizophagus irregularis* and harvested at three weeks after inoculation. Data shown are averages +/- standard deviation of three biological replicates. Transcript accumulation was normalized against a reference gene index (*Mtubi*, *MtPdf2*, *MtEf1*) (Branscheid et al. 2010). Different letters indicate fresh weight values that differ significantly from each other (P<0.05).

Figure S4

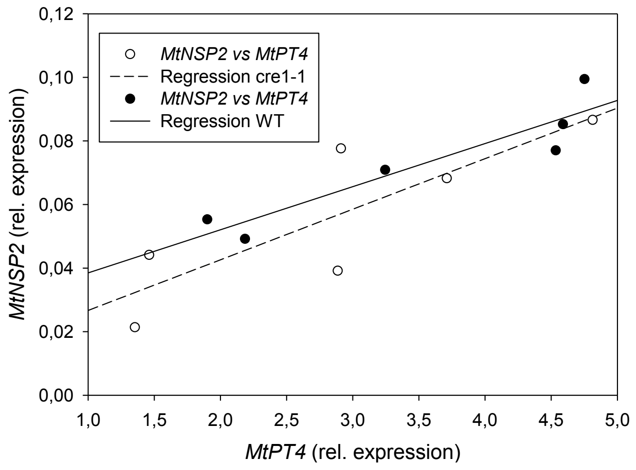

Figure S4

The *cre1-1* plant line shows a similar correlation between the relative *NSP2* and *MtPt4* transcript abundance like wild type plants.

Scatter plot of the relative expression of *NSP2* against the relative expression of *MtPt4* of individual WT plants (black dots) and *cre1-1* plants (hollow dots) including linear regression lines for each plant line. The data for the WT plants is identical to the data presented in figure 3. Both lines independently showed a statistically significant correlation ($P < 0.05$, Pearson product moment correlation). All plants were harvested 4 wpi. Normalization of the expression data was carried out against a reference gene index (*MtPdf*2 and *MtEf*1).

Figure S5

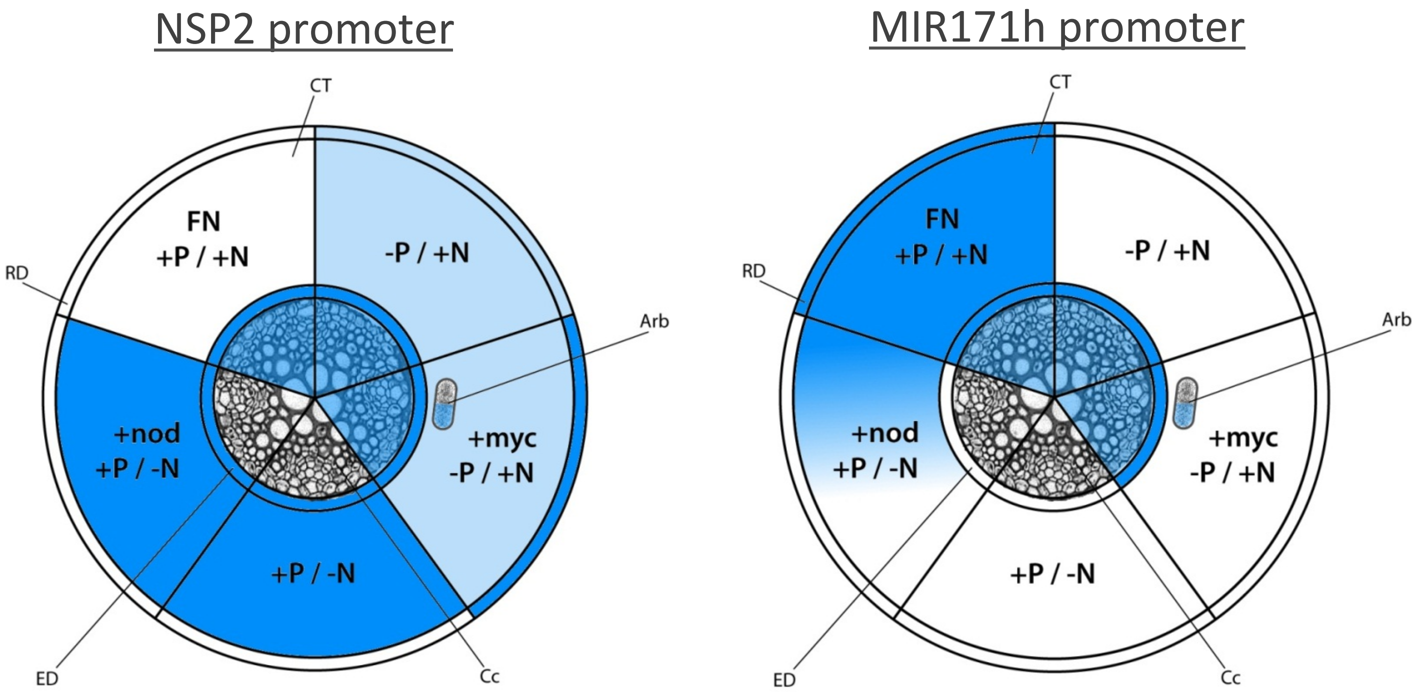

Figure S5

Cartoon representation summarizing the localization of the *NSP2* and MIR171h promoter.

The cartoon depicts the the summarized localization of promoter activities in the different tissues of the plant root shown in figure 5. Dark blue represents clear GUS signals, light blue represents faint GUS signals difficult to discern from background signal or staining artifacts. Gradient blue represents a GUS staining signal close to and flanking root nodules. FN: full nutrition, myc: mycorrhizal colonization, nod: nodulated roots, +P: 1 mM Pi, -P: 20 µM Pi, +N: 5 mM, -N: 0 mM, Arb: arbuscule, Cc: central cylinder, CT: cortex, ED: endodermis, RD: root epidermis.

Figure S6

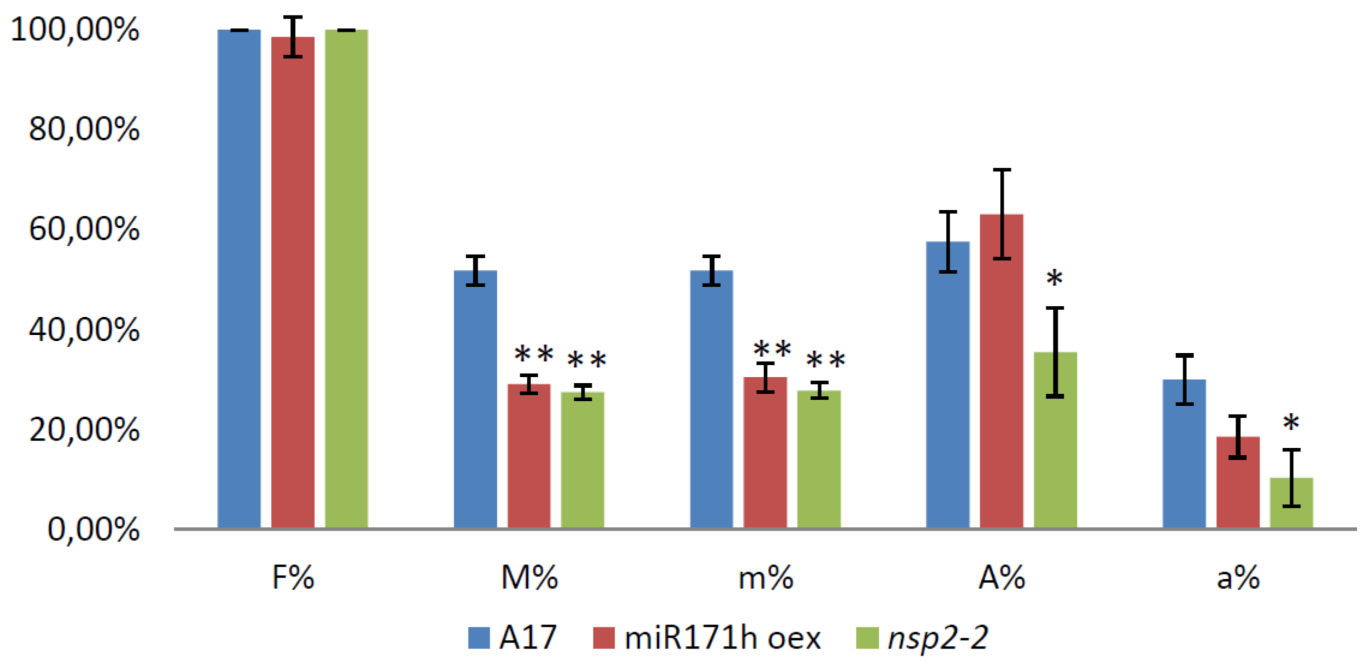

Figure S6

Mycorrhizal parameters from root transformed wild type and miR171h overexpression plants compared to *nsp2-2* plants

Mycorrhizal parameters were determined according to Trouvelot et al. (1986). Wild-type *M. truncatula* plants cv A17 (blue) and *nsp2-2* (green) plants were transformed with an empty vector control, whereas miR171h oex (red) represents wild-type plants transformed with MIR171h-GFP. The plants were harvested 4 weeks post inoculation. Error bars represent standard errors. Statistical differences compared to A17 determined by Student's t-test are represented by asterisk (*: P ≤0.05; **: P ≤ 0.01). F%: Mycorrhizal frequency in the root system, M%: Mycorrhizal intensity in the root system, m%: Mycorrhizal intensity in the root fragments, A%: Arbuscule abundance in the root system, a%: Arbuscule abundance in mycorrhizal parts of root fragments.
